# Editorial: Equity in Transplantation: A Commitment for Progress in Troubled Times

**DOI:** 10.3389/ti.2022.10781

**Published:** 2022-08-24

**Authors:** Thierry Berney, Ifeoma I. Ulasi, Chloë Balleste, Paulo N. Martins, Maria Irene Bellini, Hannah A. Valantine, Luciano Potena

**Affiliations:** ^1^ Transplant International; ^2^ Division of Transplantation, Department of Surgery, University of Geneva Hospitals, Geneva, Switzerland; ^3^ College of Medicine, University of Nigeria, Enugu, Nigeria; ^4^ Renal Unit, Department of Internal Medicine, Alex Ekwueme Federal University Teaching Hospital, Abakaliki, Nigeria; ^5^ Surgery and Surgical Specializations Department, Faculty of Medicine, University of Barcelona, Barcelona, Spain; ^6^ Donation and Transplantation Institute, DTI Foundation, Barcelona, Spain; ^7^ Department of Surgery, Transplant Division, University of Massachusetts, Worcester, MA, United States; ^8^ Department of Surgical Sciences, Sapienza University of Rome, Rome, Italy; ^9^ Department of Medicine, Stanford University School of Medicine, Palo Alto, CA, United States; ^10^ Heart Failure and Transplant Unit, IRCCS Azienda Ospedaliero-Universitaria di Bologna, Bologna, Italy

**Keywords:** diversity and inclusion, equity, discrimination, gender equality, ethnicity, transplantation

In the last few months, we have witnessed a series of events jeopardizing basic human rights in parts of the world that used to stand for them. In one of the world’s oldest democracies, essential woman’s rights, basic aspirations of the LGBT community, voting rights of underprivileged populations, and the right to equitable healthcare are being suppressed or grossly challenged. As the world attempts to recover from the disarray brought by the COVID 19 pandemic, nationalist parties targeting migrants and other minorities as scapegoats, are increasingly entering the parliaments and governments of countries with democratic traditions. As the Ukraine-Russia conflict roars in the heart of Europe, 20 more countries are experiencing significant civil wars, terrorist insurgencies, or ethnic violence (1). According to the World Bank, more than 50% of the population lives below the poverty line in 19 countries and some of the wealthiest western economies have more than 15% meeting the poverty criteria (2). We are experiencing worldwide a worrisome increase in attacks and discrimination based on gender, race, ethnicity, sexual orientation, gender identity, nationality, religion, education, and other features of diversity that characterize a human being.

Transplantation is a therapeutic strategy founded on an altruistic gift. In this troublesome context we, who are involved in transplantation, have more than ever an urgent and specific duty to safeguard the value of this gift by acting to ensure equity in the delivery of care, preserve the value of diversity and inclusion, and remove the biases that limit access to transplantation.

At Transplant International, we firmly believe that diversity is the essence of humankind and inclusion is the engine that drives and sustains the quality of our work. Whether from the transplant patient or the transplant professional standpoint, we believe that equal access to healthcare, as well as to professional development and academic career are self-evident rights, and that ensuring the implementation of these principles is the duty and responsibility of the leaders in the relative microcosm that is transplantation.

From the patient perspective, the issues to tackle are manifold and were highlighted in the call to action launched by ESOT on the occasion of its 40th anniversary (3). As a few examples among many: conditions, such as diabetes, obesity, and hepatitis B/C, are more prevalent in certain racial and ethnic groups, which negatively impacts donation and transplantation rates in disproportionately high numbers (4); patients with higher income and education have greater access to transplantation (5); immigrants face barriers in access to transplant services, including lower awareness and a lack of full healthcare coverage (6); women donate more organs than they receive, while men making up the majority of organ transplant recipients, in particular, because of psychological and socio-economic factors (7); there are significant regional and national variations in the number of transplants performed. In many countries, transplant centers are not evenly distributed, in favor of wealthier areas. This is even more critical in emerging economies, let alone least developed countries, where access to transplantation is often non-existing.

The devil can sometimes hide in the details. A universal and easy measure of kidney function, the eGFR (estimated glomerular filtration rate) has been calculated for decades with a modifier for “black people,” introducing in effect a bias leading to systemic underestimation of kidney disease severity in black patients and delaying their access to kidney transplantation (8). The board of directors of the US Organ Procurement and Transplantation Network (OPTN) has very recently (June 2022) abolished the modifier for black people in the calculation of eGFR, in a commendable effort to remove one of the obstacles to timely kidney transplantation in a population disproportionately affected by end-stage kidney disease (8). This specific issue will be reviewed in more detail in Transplant International in the near future.

Regarding professional careers, the field of transplantation does not fare any better than other fields in medicine and medical sciences. A recent survey revealed alarmingly high rates of ethnic and gender disparity, lack of mentorship, and very low rates of female leadership in the liver transplantation field (9); in terms of first and senior authorship, gender disparity has improved over the past 20 years, but is still blatantly obvious (10); finally, the editorial boards of journals, including in the field of transplantation, still have gross imbalances in their compositions in terms of gender and ethnic equity (11-13).

As stated in the Transplant International website, “we value engagement and inclusion at all stages of science communication and dissemination, from the submission of research manuscripts, through the editorial and review process and on to publication.” The gender-balanced editorial board (14) “welcomes submissions from applicants of all ethnicities, nationalities, religions, gender identities, sexual orientations or other individual status, and are committed to eliminating the influence of any bias in our processes.” To bring this commitment further, and following the call for action launched at the opening ceremony of the 2021 ESOT congress in Milan and the mandate of the ESOT Action Day announced at the celebration of ESOT 40th anniversary (3), Transplant International is pleased to announce the launching of a Special issue on “Diversity, Equity and Inclusion in Transplantation” (15).

The scope of this issue is not only to highlight the problems currently limiting inclusion and equity in transplantation, but to propose evidence-based solutions, that could guide changes in policies and practices. We encourage all members of the transplantation community to show their commitment to this far-reaching cause and contribute to this endeavour.
